# Development of a triplex real-time quantitative PCR for detection and differentiation of genotypes I and II African swine fever virus

**DOI:** 10.3389/fvets.2023.1278714

**Published:** 2023-10-19

**Authors:** Xinxiu Qian, Liping Hu, Kaichuang Shi, Haina Wei, Yuwen Shi, Xin Hu, Qingan Zhou, Shuping Feng, Feng Long, Shenglan Mo, Zongqiang Li

**Affiliations:** ^1^College of Animal Science and Technology, Guangxi University, Nanning, China; ^2^Guangxi Center for Animal Disease Control and Prevention, Nanning, China

**Keywords:** African swine fever virus (ASFV), genotype I, genotype II, B646L gene, E183L gene, F1055L gene, multiplex real-time quantitative PCR (multiplex qPCR)

## Abstract

African swine fever virus (ASFV) was first identified in 1921 and is extensively prevalent around the world nowadays, which has a significant negative impact on the swine industry. In China, genotype II ASFV was first discovered in 2018, and has spread quickly to different provinces in a very short time; genotype I ASFV was first found in 2020, and has been reported in several provinces since then. To establish an accurate method for detection and differentiation of genotypes I and II ASFV, three primers and probes were designed targeting the ASFV B646L gene for different genotypes, the F1055L gene for genotype I, and the E183L gene for genotype II, and a triplex real-time quantitative PCR (qPCR) for differential detection of genotypes I and II ASFV was developed after optimizing the reaction conditions. The assay showed high sensitivity, and the limits of detection (LOD) of the B646L, F1055L, and E183L genes were 399.647 copies/reaction, 374.409 copies/reaction, and 355.083 copies/reaction, respectively; the coefficients of variation (CVs) of the intra-assay and the inter-assay were 0.22–1.88% and 0.16–1.68%, respectively, showing that this method had good repeatability; the assay could detect only ASFV, without cross-reactivity with other swine viruses including PRRSV, PEDV, PDCoV, CSFV, PRV, and PCV2, showing excellent specificity of this method. A total of 3,519 clinical samples from Guangxi province, southern China, were tested by the developed assay, and 8.16% (287/3,519) samples were found to be positive for ASFV, of which 0.17% (6/3,519) samples were positive for genotype I, 7.19% (253/3,519) samples for genotype II, and 0.80% (28/3,519) samples for genotypes I and II. At the same time, these clinical samples were also tested by a previously reported multiplex qPCR, and the agreement between these two methods was more than 99.94%. In summary, the developed triplex qPCR provided a fast, specific and accurate method for detection and differentiation of genotypes I and II ASFV.

## 1. Introduction

African swine fever virus (ASFV) is a double-stranded DNA virus with a genome about 170 kb–193 kb, which encodes 68 structural proteins and more than 100 non-structural proteins (1). ASFV belongs to the genus *Asfivirus* in the family *Asfarviridae*, and causes highly contagious disease in infected domestic pigs and wild boars (2), which is characterized by high fever, lethargy, skin cyanosis, and extensive haemorrhage in visceral organs, with high rate of morbidity up to 100% (3). African swine fever (ASF) broke out in China in 2018, and has since then reported in many countries in Asia, including South Korea, Vietnam, Laos, Cambodia, and Indonesia (4, 5). ASF was also reported in domestic pigs on the island of Hispaniola (Haiti and the Dominican Republic) in North America in 2021 (6), and in domestic pigs and wild boars in Europe in 2014 (7). The pig industry has suffered huge economic losses due to ASF (8–10).

ASFV can be divided into 24 genotypes based on the B646L gene, which encodes the p72 protein (11). Most genotypes of ASFV are mainly prevalent in the sub-Saharan Africa, and only genotypes I and II have been reported within and outside Africa (12, 13). In 2007, genotype II ASFV first spread to Georgia, then spread to many countries and regions in Europe and North America. The first outbreak of genotype II ASF was reported in China in 2018 (6), and then it was found in South Korea, Vietnam, Laos, Cambodia, Indonesia, and other Asian countries (6, 7). Genotype I ASFV was first found in Portugal outside Africa in 1957, and appeared in some countries in Europe and North America (14). In 2020, genotype I ASFV was first discovered in pig farms in China (15, 16). Since genotype II ASFV were first discovered in China, these strains have spread rapidly to most provinces, resulting in a nationwide outbreak of ASF, which has caused serious damage to the Chinese swine industry (17, 18). Furthermore, genotype I strains of ASFV have been discovered in several provinces (Anhui, Shandong, and Guangxi provinces) in China since 2020 (15, 16, 19), and the prevalence situation of genotype I ASFV and its economic losses to the pig industry still needed to be evaluated. Therefore, it is very important to differentially detect genotypes I and II ASFV in the early stage of infection in order to master the infection and epidemic situation, and take effective measures for prevention and control of this disease.

The real-time quantitative PCR (qPCR) is a convenient, efficient, sensitive, accurate and high throughput technology, and has been widely used to detect viral nucleic acids (20, 21). To date, the qPCR has been developed for detection and diagnosis of ASFV (22–26). Several multiplex qPCR based on the E296R, B646L, or E183L genes of ASFV have been reported for the detection of genotypes I and II ASFV in 2022 (27–29). Li et al. used two pairs of primers and probes based on the E296R gene to detect genotypes I and II ASFV, respectively (27); Cao et al. designed a pair of primers and two probes based on the B646L gene to differentiate genotypes I and II ASFV (28); Gao et al. designed one pair of primers and probe based on the B646L gene to detect both genotypes I and II ASFV, and another pair of primers and probe based on the B183L gene to detect genotype I ASFV (29). In this study, a triplex qPCR based on the ASFV B646L, F1055L, and E183L genes was developed for detection and differentiation of genotypes I and II ASFV. The primers and probe targeting the B646L gene were used as the universal primers and probe to detect different genotypes of ASFV, while the primers and probes targeting the F1055L gene, and the E183L gene were used to detect genotype I, and genotype II ASFV, respectively. The developed triplex qPCR showed high specificity, sensitivity, and repeatability to differentiate genotypes I and II ASFV.

## 2. Materials and methods

### 2.1. Design of primers and probes

**Figure 1:**
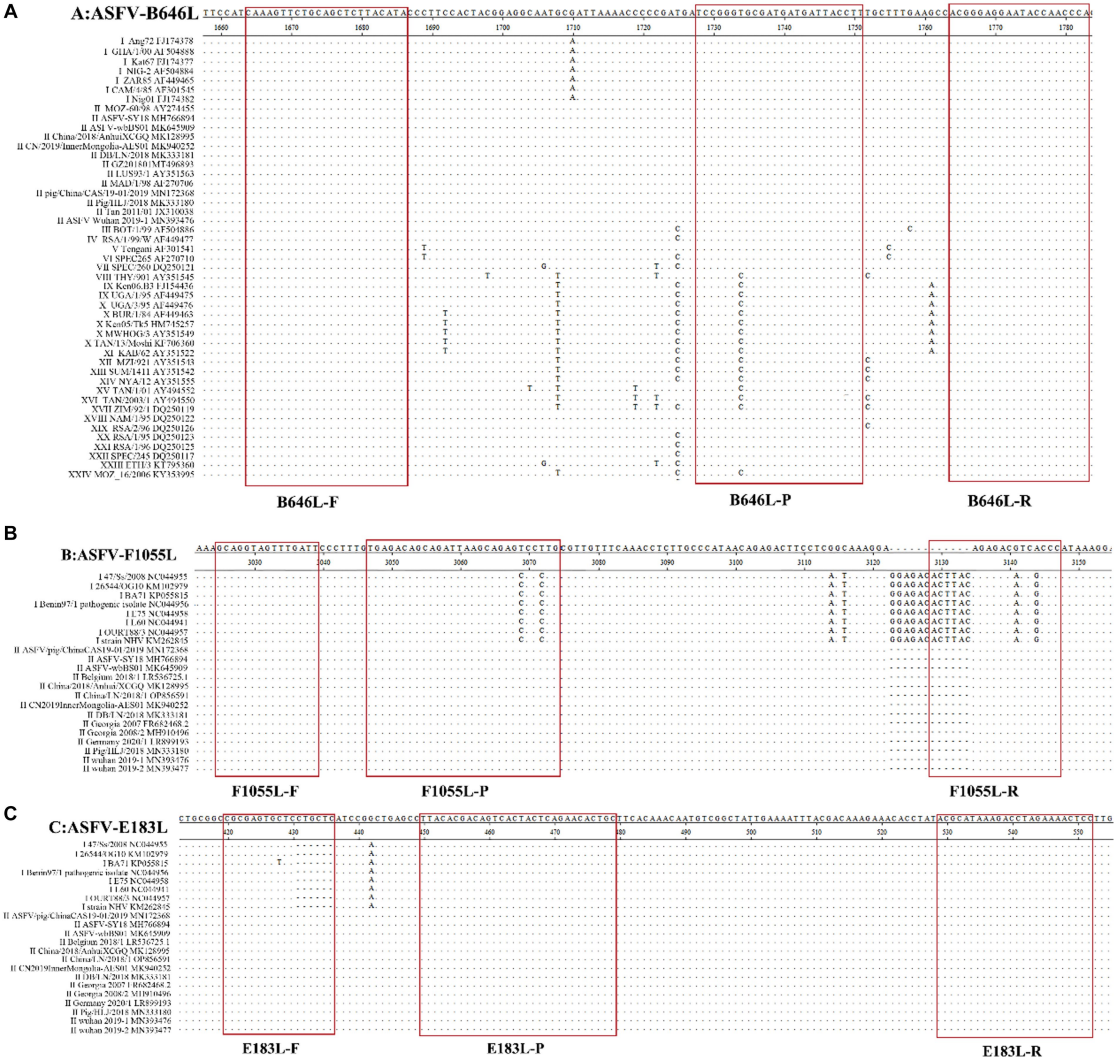
The locations of the specific primers and probes designed for the triplex qPCR. The nucleotide sequence alignments of the partial B646L gene **(A)**, F1055L gene **(B)**, and E183L gene **(C)** of ASFV show the locations of the primers and probes. F, P, and R indicate the forward primer, TaqMan probe, and reverse primer, respectively. “·” indicates the same nucleotide acid and “-” indicates the deletion of nucleotide acid.

The specific pair of primers and corresponding probe were designed targeting the highly conserved region of the B646L gene to simultaneously detect different genotypes of ASFV strains. The primers and probes were designed targeting the F1055L gene, and the E183L gene to specifically detect genotype I, and genotype II ASFV strains, respectively (Table 1). The purpose of the assay is to determine whether ASFV is present in the clinical sample, and if so, it will be further determined whether the strain belongs to genotype I and/or genotype II ASFV. The specificity of primers and probes were confirmed by GenBanK BLAST analysis.^1^ The location of the primers and probes were indicated in Figure 1.

**Table 1:** The designed primers and probes.

Name	Sequence (5′ → 3′)	Tm/°C	Product/bp
B646L-F	CAAAGTTCTGCAGCTCTTACA	56.0	120
B646L-R	TGGGTTGGTATTCCTCCCGT	61.6	
B646L-P	FAM-TCCGGGYGCGATGATGATTACCTT-BHQ1	63.1	
F1055L-F	GCAGGTAGTTTGATTCCCTT	56.0	122
F1055L-R	GGGCGATGTCTCTGTAAGT	57.6	
F1055L-P	VIC-TGAGACAGCAGATTAAGCAGAGCCCCTG-BHQ1	67.4	
E183L-F	CGCGAGTGCTCCTGCTC	60.1	133
E183L-R	GGAGTTTTCTAGGTCTTTATGCGT	57.6	
E183L-P	CY5-TTACACGACAGTCACTACTCAGAACACTGC-BHQ2	64.0	

Y = C/T.

To verify the primers and probe ASFV-B646L-F/R/P could detect different genotypes of ASFV, the nucleotide sequences of the targeting region of 24 genotypes of ASFV were aligned, and confirmed that the primers and probe were located in the conserved region (Figure 1). Furthermore, the nucleotide sequences of the targeted fragments of the 24 genotypes were synthesized (Supplementary Table S1), and transformed into plasmids. The recombinant plasmids were used as templates to verify that the primers and probe ASFV-B646L-F/R/P could specifically detect these 24 genotypes of ASFV.

### 2.2. Viral strains and clinical samples

The vaccine strains of classical swine fever virus (CSFV, C strain), porcine reproductive and respiratory syndrome virus (PRRSV, JXA1-R strain), foot-and-mouth disease virus (FMDV, O/Mya98/XJ/2010 strain), and pseudorabies virus (PRV, Bartha-K61 strain) were bought from the China Animal Husbandry Industry Co. Ltd. (Beijing, China). The vaccine strains of swine influenza virus (SIV, TJ strain), porcine epidemic diarrhea virus (PEDV, CV777 strain), and porcine circovirus type 2 (PCV2, ZJ/C strain) were bought from the Huapai Biological Group Co. Ltd. (Chengdu, China). The positive samples of genotype I, and genotype II ASFV were provided by Guangxi Center for Animal Disease Control and Prevention (CADC), China. The vaccines and samples were stored at −80°C until used.

A total of 3,159 clinical samples, including whole blood, and the tissues of liver, spleen, lung, kidney, and lymph nodes from each pig (The pooled clinical tissue homogenate of liver, spleen, lung, kidney, and lymph nodes from each pig was considered as one sample when tested by the multiplex qPCR), were collected from 3,159 pigs in different pig farms, harmless disposal sites, slaughterhouses, and farmers markets in Guangxi province, southern China from March 2019 to February 2023. The pigs from pig farms, slaughterhouses, and farmers markets were clinically healthy pigs, and the pigs from harmless disposal sites were abnormal deaths of pigs with different manifestations. Before collecting the samples, the consent of the animal owners had been obtained to use the samples for this study. The samples were stored at −80°C until used.

### 2.3. Extraction of viral genomic DNA

The pooled clinical tissue homogenates (20%, W/V) were resuspended in phosphate-buffered saline (PBS, pH 7.2), freeze-thawed three times, vortexed, centrifuged at 12,000 × g at 4°C for 5 min, and 200 μL of supernatant was used to extract the total nucleic acids.

The anticoagulated whole blood samples containing EDTA-Na_2_ (15 g/L) were spun for 10 s, centrifuged at 12,000 × g for 5 min at 4°C, and 200 μL of supernatant was used to extract the total nucleic acids.

The total nucleic acids were extracted by the GeneRotex 96 Automated Nucleic Acid Extractor (Tianlong, Xi’an, China) using the Viral DNA/RNA Isolation Kit 4.0 (Tianlong, Xi’an, China) following the manufacturer’s instructions. The total nucleic acids were stored at −80°C until used.

### 2.4. Construction of the standard control plasmid constructs

The total nucleic acids from the positive samples of genotype I, and genotype II ASFV were amplified by PCR using the specific primers (Table 1). The products of the B646L, F1055L, and E183L genes were purified, and used to generate the standard control plasmid constructs according to Liu et al. (16). The recombinant standard plasmid constructs were confirmed by sequence analysis, and named pASFV-B646L, pASFV-F1055L, and pASFV-E183L, respectively. The ultraviolet absorbance of the standard plasmid constructs at 260 nm and 280 nm was measured, and the following formula was used to determine their concentrations: $\mathrm{plasmid}\;\left(\mathrm{copies}/\mathrm{\mu L}\right)$ = $\frac{\left(6.02\times 10^{23}\right)\times \left(X\;\mathrm{ng}/\mathrm{\mu L}\times 10^{-9}\right)}{\mathrm{plasmid}\;\mathrm{length}\left(\mathrm{bp}\right)\times 660}$ .

### 2.5. Optimization of the reaction conditions

The QuantStudio 6 qPCR system (ABI, Carlsbad, CA, United States) was used to develop the triplex qPCR. The amplification conditions of 95°C for 30 s, 40 cycles of 95°C for 5 s, and 58°C for 30 s were used to determine the annealing temperature and the concentrations of the primers and probes. The reaction system in a total volume of 20 μL included Premix Ex Taq (Probe qPCR) (TaKaRa, Beijing, China), three pairs of the primers and probes, the mixtures of the three standard plasmid constructs, and distilled water. Different annealing temperatures (56°C, 57°C, 58°C, 59°C, 60°C) were used for amplification, different concentrations of primers and probes was adjusted by the matrix method, and the optimal reaction conditions were determined.

### 2.6. Generation of the standard curves

The three standard plasmid constructs pASFV-B646L, pASFV-F1055L, and pASFV-E183L were mixed at a ratio of 1:1:1, then 10-fold serially diluted from 1.55 × 10^8^ to 1.55 × 10^2^ copies/μL (final concentration in the reaction system: 1.55 × 10^7^ to 1.55 × 10^1^ copies/μL), and used as templates to generate the standard curves.

### 2.7. Evaluation of the analytical specificity

Analytical specificity refers to the ability of an assay to measure one particular organism or substance, rather than others, in a sample. This parameter in qPCR refers to the specificity of primers for target of interest. For evaluation of the analytical specificity of the developed triplex qPCR in this study, the total nucleic acids (RNA/DNA) of PRRSV, PEDV, FMDV, CSFV, SIV, ASFV, PRV, and PCV2 were extracted from vaccine solution or positive clinical samples using the Viral DNA/RNA Isolation Kit 4.0 (Tianlong, Xi’an, China) following the manufacturer’s instructions. The RNA of PRRSV, PEDV, FMDV, CSFV, and SIV was reverse transcribed to cDNA using PrimeScript II 1st Strand cDNA Synthesis Kit (TaKaRa, Beijing, China) according to the manufacturer’s instructions, respectively. The DNA of ASFV, PRV, and PCV2 was used to analyze the specificity of the assay directly.

To analyze the specificity of the developed triplex qPCR, the DNA of ASFV, PRV, and PCV2, and the cDNA of PRRSV, PEDV, FMDV, CSFV, and SIV were used as templates, and the distilled water was used as negative control.

### 2.8. Evaluation of the analytical sensitivity

Analytical sensitivity (Limit of Detection, LOD) represents the smallest amount of substance in a sample that can accurately be measured by an assay. The LOD in qPCR is defined as the spike amount of target organism in dilution that can be detected in 95% of replicates. For evaluation of the analytical sensitivity of the developed triplex qPCR in this study, the three standard plasmid constructs pASFV-B646L, pASFV-F1055L, and pASFV-E183L were mixed at a ratio of 1:1:1, then 10-fold serially diluted from 1.55 × 10^8^ to 1.55 × 10^0^ copies/μL (final concentration in the reaction system: 1.55 × 10^7^ to 1.55 × 10^−1^ copies/μL), and used as templates to determine the LOD of each plasmid. The Probit regression was used to analyze the LOD of the developed triplex qPCR.

### 2.9. Evaluation of the repeatability

The three standard plasmid constructs pASFV-B646L, pASFV-F1055L, and pASFV-E183L were mixed at a ratio of 1:1:1, then 10-fold serially diluted. Three concentrations of 1.55 × 10^7^, 1.55 × 10^5^, and 1.55 × 10^3^ copies/μL (the final reaction concentration) were used as templates to evaluate the intra-assay variation and the inter-assay variation. The intra-assay was repeated in three times, and the inter-assay was done on three different days, and the coefficients of variation (CVs) were determined.

### 2.10. Detection of the clinical samples

The established triplex qPCR was used to test 3,519 clinical samples from 3,519 pigs in Guangxi province, southern China. At the same time, these samples were also tested by the duplex qPCR developed by Cao et al. (28), and the agreement of both methods were analyzed using Chi-square-test analysis.

In addition, the clinical and diagnostic sensitivity refers to a measure of a test’s ability to recognize positives in a sample, and the clinical and diagnostic specificity refers to a measure of a test’s ability to recognize negatives in a sample. The clinical and diagnostic sensitivity, and specificity are used to compare novel qPCR methods with reference methods. In this study, the clinical sensitivity and clinical specificity of the developed triplex qPCR was determined by comparing the detection results of the clinical samples using this assay with those detection results of the clinical samples using the reference method (28).

To further validate the detection results of the developed assay, all the samples were also tested by the singleplex qPCR for detection of ASFV described in the Chinese national standard: *Diagnostic techniques for African swine fever* (GB/T 18648-2020).^2^

## 3. Results

### 3.1. Construction of the standard control plasmid constructs

The B646L, F1055L, and E183L genes of ASFV were amplified by PCR, and the products were used to generate the standard control plasmid constructs. The concentrations of the three standard plasmid constructs named pASFV-B646L, pASFV-F1055L, and pASFV-E183L were determined to be 2.35 × 10^10^, 1.55 × 10^10^, and 2.08 × 10^10^ copies/μL (original concentration), respectively. The three standard plasmid constructs were diluted to 1.55 × 10^10^ copies/μL, and stored at −80°C until used.

**Figure 2:**
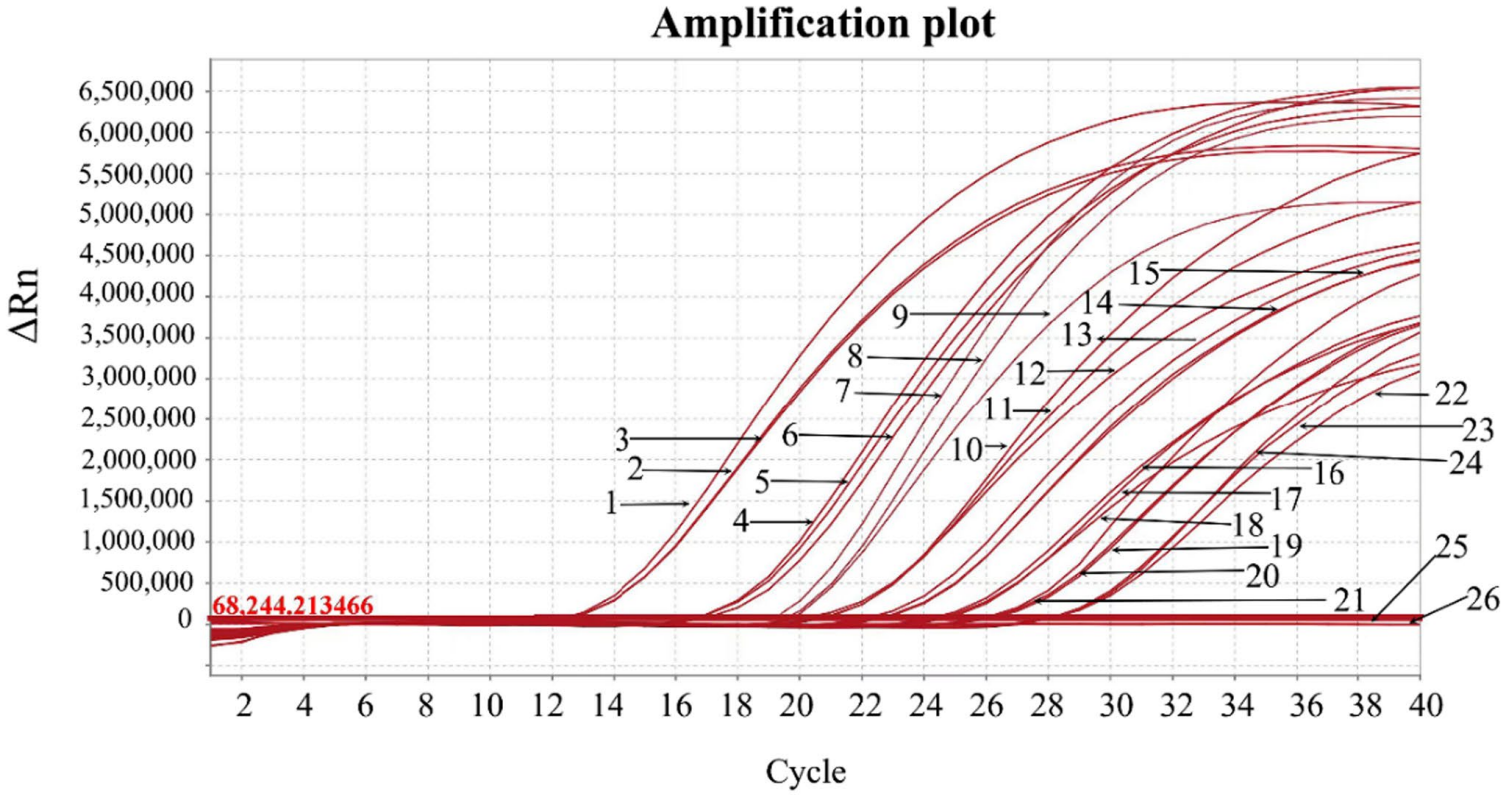
The amplification curves of the recombinant plasmid constructs generated for 24 genotypes of ASFV. The recombinant plasmid constructs were used as templates to validate the specific primers and probe ASFV-B646L-F/R/P. 1: pASFV-I; 2: pASFV-II; 3: pASFV-III; 4: pASFV-IV; 5: pASFV-V; 6: pASFV-VI; 7: pASFV-VII; 8: pASFV-VIII; 9: pASFV-IX; 10: pASFV-X; 11: pASFV-XI; 12: pASFV-XII; 13: pASFV-XIII; 14: pASFV-XIV; 15: pASFV-XV; 16: pASFV-XVI; 17: pASFV-XVII; 18: pASFV- XVIII; 19: pASFV-XIX; 20: pASFV-XX; 21: pASFV-XXI; 22: pASFV-XXII; 23: pASFV-XXIII; 24: pASFV-XXIV; 25: negative clinical sample control; 26: RNase-free distilled water control.

The primers and probe ASFV-B646L-F/R/P (Table 1) were used to detect the recombinant plasmid constructs with the target fragments of 24 genotypes of ASFV. The results showed that all 24 genotypes were detected by ASFV-B646L-F/R/P (Figure 2), indicating that ASFV-B646L-F/R/P could be used as universal primers and probe to specifically detect different genotypes of ASFV.

### 3.2. Determination of the optimal reaction conditions

After optimizing different annealing temperatures, primer and probe concentrations, the developed triplex qPCR in a total volume of 20 μL included Premix Ex Taq (TaKaRa, Beijing, China), ASFV-B646L-F/R/P, ASFV-F1055L-F/R/P, ASFV-E183L-F/R/P, total nucleic acids, and RNase-free distilled water (Table 2). The amplification procedure was: 95°C for 30 s, followed by 40 cycles of 95°C for 5 s and 58°C for 30 s. At the end of each cycle, the fluorescence signals were recorded. The sample with a Ct value ≤36 was considered as positive sample.

**Table 2:** The reaction system for the triplex qPCR.

Reagent	Volume (μL)	Final concentration (nM)
Premix Ex Taq (2×)	10	/
ASFV-B646L-F (20 μM)	0.3	300
ASFV-B646L-R (20 μM)	0.3	300
ASFV-B646L-P (20 μM)	0.3	300
ASFV-F1055L-F (20 μM)	0.3	300
ASFV-F1055L-R (20 μM)	0.3	300
ASFV-F1055L-P (20 μM)	0.2	200
ASFV-E183L-F (20 μM)	0.4	400
ASFV-E183L-R (20 μM)	0.4	400
ASFV-E183L-P (20 μM)	0.2	200
Total nucleic acids	2.0	/
RNase-free distilled water	Up to 20	/

### 3.3. Generation of the standard curves

**Figure 3:**
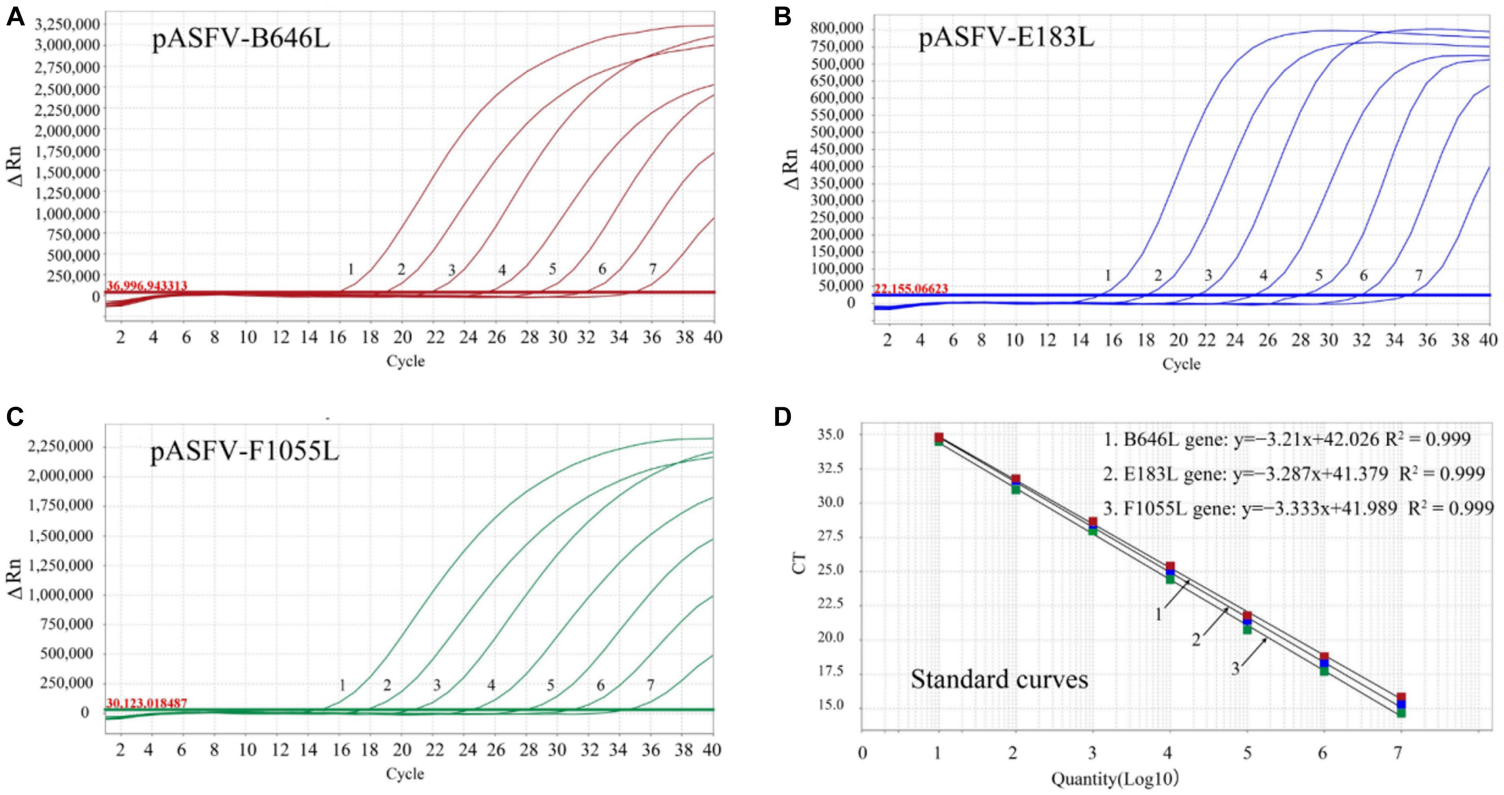
Generation of the standard curves. The amplification curves of pASFV-B646L **(A)**, pASFV-E183L **(B)**, pASFV-F1055L **(C)**, and their standard curves **(D)** are shown. 1–7: the final concentrations of the standard plasmid constructs ranged from 1.55 × 10^7^ to 1.55 × 10^1^ copies/μL.

The three standard plasmid constructs pASFV-B646L, pASFV-F1055L, and pASFV-E183L were 10-fold serially diluted from 1.55 × 10^8^ to 1.55 × 10^2^ copies/μL (final concentration in the reaction system: 1.55 × 10^7^ to 1.55 × 10^1^ copies/μL), and used to generate the standard curves of the developed triplex qPCR. The amplification efficiencies, slopes, and correlation coefficients (*R*^2^) were 104.9%, −3.21, and 0.999 for the B646L gene; 99.63%, −3.333, and 0.999 for the F1055L gene; 101.5%, −3.287, and 0.999 for the E183L gene (Figure 3).

### 3.4. Specificity analysis

**Figure 4:**
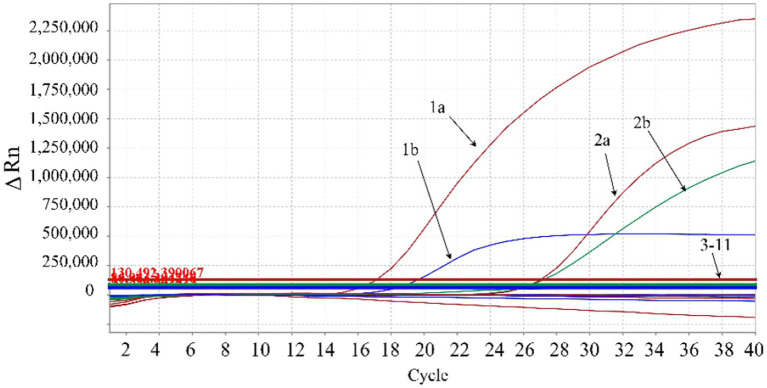
Specificity analysis. 1a: ASFV genotype II B646L gene; 1b: ASFV genotype II E183L gene; 2a: ASFV genotype I B646L gene; 2b: ASFV genotype I F1055L gene; 3–9: PRRSV, PEDV, FMDV, CSFV, SIV, PRV, PCV2, respectively; 10: negative clinical sample control; 11: RNase-free distilled water control. In the figure, the red amplification curves correspond to primers and probe targeting ASFV B646L gene, the blue amplification curve corresponds to the primers and probe targeting ASFV E183L gene, and the green amplification curve corresponds to the primers and probe targeting ASFV F1055L gene.

The DNA/cDNA of genotypes I and II ASFV, PRRSV, PEDV, FMDV, CSFV, SIV, PRV, PCV2, and RNase-free distilled water were used as templates for amplification. The results showed that there were amplification curves of genotypes I and II ASFV, while no amplification curve was obtained from other swine viruses, indicating good specificity of the developed triplex qPCR without cross-reactivity with other viruses (Figure 4).

### 3.5. Sensitivity analysis

**Figure 5:**
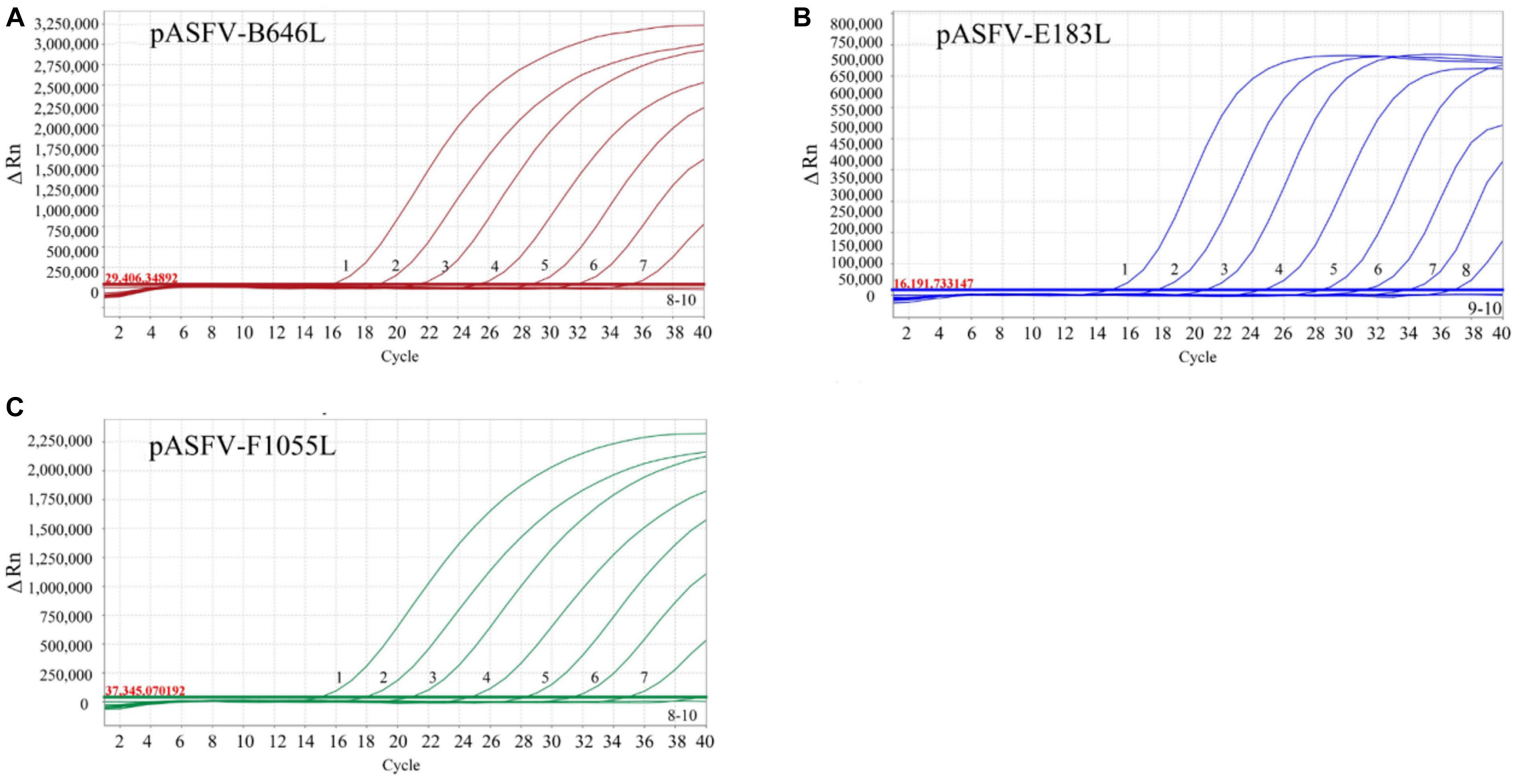
Sensitivity analysis. 1–9: the final concentrations of the standard plasmid constructs pASFV-B646L **(A)**, pASFV-E183L **(B)**, and pASFV-F1055L **(C)** ranged from 1.55 × 10^7^ to 1.55 × 10^−1^ copies/μL. 10: negative control (RNase-free distilled water).

The three standard plasmid constructs pASFV-B646L, pASFV-F1055L, and pASFV-E183L were 10-fold serially diluted from 1.55 × 10^8^ to 1.55 × 10^0^ copies/μL (final concentration in the reaction system: 1.55 × 10^7^ to 1.55 × 10^−1^ copies/μL), and used to evaluate the LOD of each plasmid construct. The results showed that the LOD of pASFV-B646L, pASFV-F1055L, and pASFV-E183L were 1.55 × 10^1^ copies/μL (the final reaction concentration), respectively (Figure 5).

**Figure 6:**
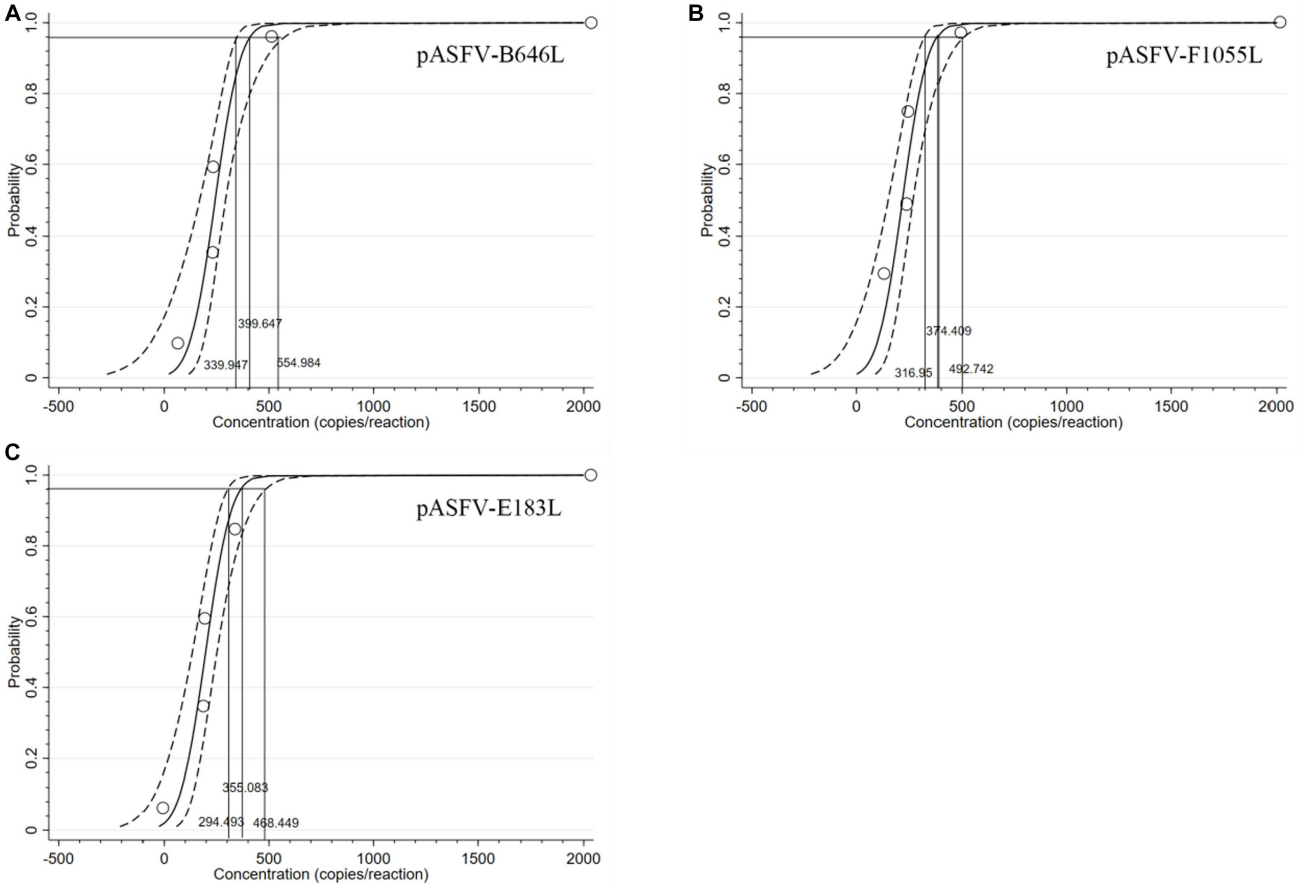
PROBIT regression results to determine the analytical sensitivity. Ct values and hit rates were measured using ten-fold serial dilutions of standard plasmid constructs. The LODs of pASFV-B646L **(A)**, pASFV-F1055L **(B)**, and pASFV-E183L **(C)** were determined to be 399.647 copies/reaction (95% confidence interval 339.947–554.984 copies/reaction), 374.409 copies/reaction (95% confidence interval 316.95–492.742 copies/reaction), and 355.083 copies/reaction (95% confidence interval 294.493–468.449 copies/reaction), respectively.

The LOD of the developed triplex qPCR was determined by testing serial dilutions of the standard plasmid constructs. Four serial dilutions of pASFV-B646L, pASFV-F1055L, and pASFV-E183L were used as templates to measure the Ct values and hit rates, and the results were shown in Table 3. The LODs of pASFV-B646L, pASFV-F1055L, and pASFV-E183L were analyzed using the PROBIT regression, and were determined to be 399.647 copies/reaction (95% confidence interval 339.947–554.984 copies/reaction), 374.409 copies/reaction (95% confidence interval 316.950–492.742 copies/reaction), and 355.083 copies/reaction (95% confidence interval 294.493–468.449 copies/reaction), respectively (Figure 6).

**Table 3:** Threshold cycle (Ct) values and hit rates of serially diluted plasmids.

Plasmid construct	Copies/reaction	Number of samples	Triplex qPCR	
			Ct (average)	Hit rate (%)
pASFV-B646L	31,000	24	28.7	100
	3,100	24	32.3	100
	310	24	35.6	75
	31	24	ND	0
pASFV-F1055L	31,000	24	28.8	100
	3,100	24	32.1	100
	310	24	35.8	83.3
	31	24	ND	0
pASFV-E183L	31,000	24	28.3	100
	3,100	24	32	100
	310	24	34.8	91.7
	31	24	ND	0

### 3.6. Repeatability analysis

Three standard plasmid constructs with 1.55 × 10^7^, 1.55 × 10^5^, and 1.55 × 10^3^ copies/μL (the final reaction concentration) were used as templates to evaluate the repeatability. The results showed that the coefficients of variation (CVs) of the intra-assay variation and the inter-assay variation were 0.22–1.88% and 0.16–1.68%, respectively (Table 4).

**Table 4:** Repeatability analysis of the triplex qPCR.

Plasmid construct	Concentration (copies/μL)	Ct Values of intra-assay			Ct Value of inter-assay		
		$\overset{-}{X}$	SD	CV (%)	$\overset{-}{X}$	SD	CV (%)
pASFV-B646L	1.55 × 10^7^	15.83	0.03	0.22	15.83	0.06	0.39
	1.55 × 10^5^	21.63	0.12	0.57	21.31	0.16	0.73
	1.55 × 10^3^	28.57	0.54	1.88	27.78	0.18	0.64
pASFV-F1055L	1.55 × 10^7^	15.46	0.04	0.26	15.14	0.26	1.68
	1.55 × 10^5^	21.39	0.10	0.47	21.52	0.03	0.16
	1.55 × 10^3^	28.61	0.35	1.22	28.07	0.13	0.45
pASFV-E183L	1.55 × 10^7^	15.35	0.07	0.47	15.15	0.21	1.37
	1.55 × 10^5^	21.38	0.06	0.27	21.34	0.06	0.28
	1.55 × 10^3^	28.52	0.49	1.72	27.91	0.05	0.16

### 3.7. Detection results of the clinical samples

**Figure 7:**
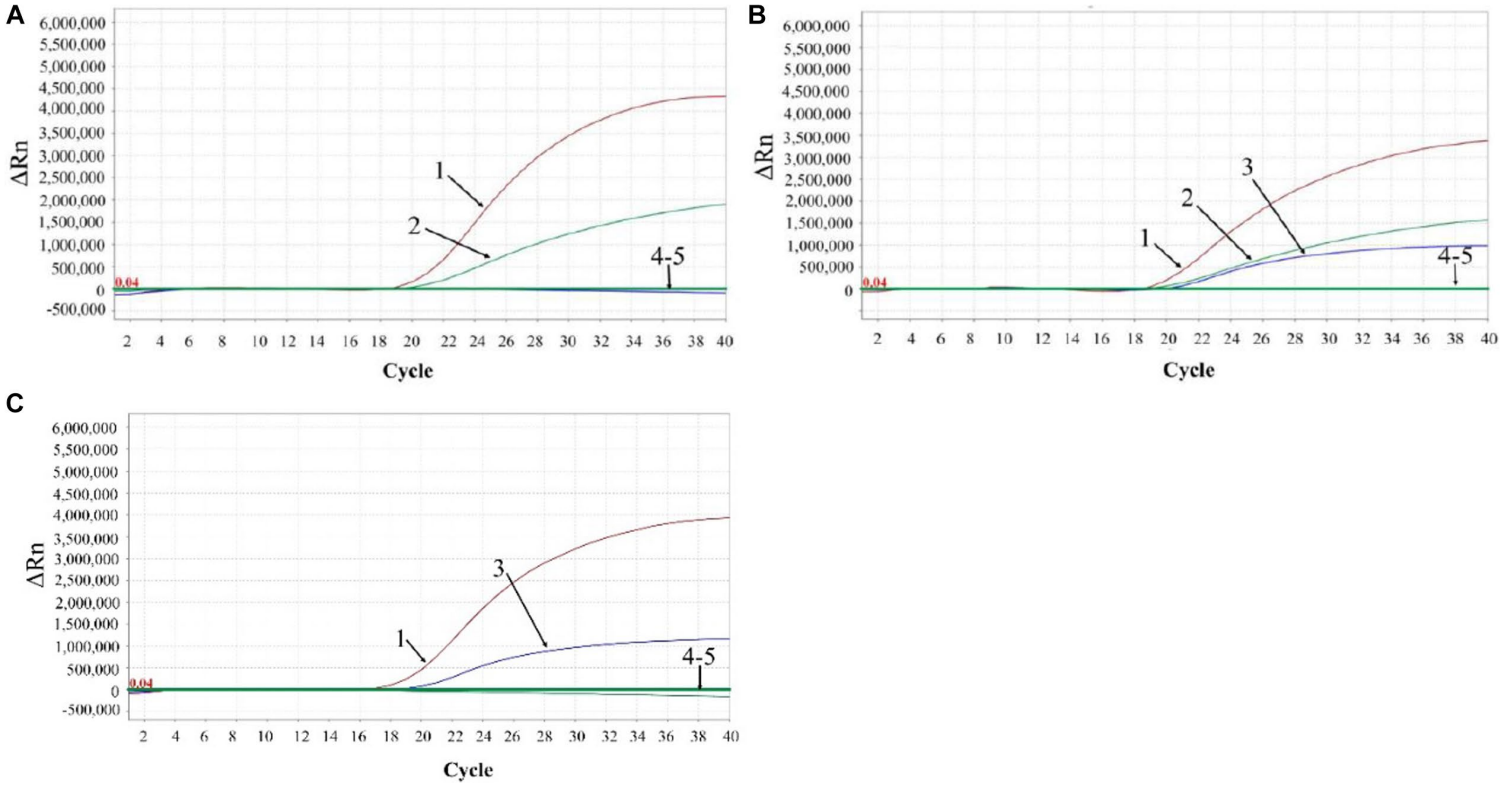
The representative amplification curves of ASFV genotype I positive sample **(A)**, genotype II positive sample **(C)**, and mixture of genotypes I and II positive sample **(B)**. 1: ASFV B646L gene; 2: ASFV F1055L gene; 3: ASFV E183Lgene; 4: negative clinical sample control; 5: RNase-free distilled water control.

A total of 3,519 clinical samples from Guangxi province in China were tested by the developed triplex qPCR. The results showed that 287 (8.16%) samples were positive for ASFV, of which 6 (0.17%), 253 (7.19%), 28 (0.80%) samples were positive for genotype I, genotype II, and both genotypes I and II, respectively (Table 5). The representative amplification curves of ASFV genotype I positive sample, genotype II positive sample, and co-infection of genotypes I and II positive sample were shown in Figure 7.

**Table 5:** The detection results of the clinical samples.

Source	Total	Positive	Genotype I	Genotype II	Genotype I and II	Positive rate
Pig farm	197	2	0	0	2	1.02% (2/197)
Harmless disposal site	906	235	3	214	18	25.72% (235/906)
Slaughterhouse	2,326	35	3	24	8	1.50% (35/2,326)
Farmers market	90	15	0	15	0	16.67% (15/90)
Total	3,519	287 (8.16%)	6 (0.17%)	253 (7.19%)	28 (0.80%)	8.16% (287/3,519)

These 3,519 samples were also tested by the qPCR established by Cao et al. (28), and showed that the positivity rates of ASFV, genotype I, genotype II, and both genotypes I and II were 8.13% (286/3,519), 0.20% (7/3,519), 7.13% (251/3,519), and 0.80% (28/3,519), respectively. Compared to the detection results of the reported reference method (28), the developed triplex qPCR showed a clinical sensitivity of 100% (98.67–100% at 95% confidence interval), and a clinical specificity of 99.97% (99.83–100% at 95% confidence interval) (Table 6).

**Table 6:** Clinical sensitivity and specificity of the developed triplex qPCR.

Triplex qPCR	The Reported Reference Method (28)		Total
	Positive	Negative	
Positive	286	1	287
Negative	0	3,232	3,232
Total	286	3,233	3,519

Clinical sensitivity: 100% (98.67–100% at 95% confidence interval). Clinical specificity: 99.97% (99.83–100% at 95% confidence interval).

Agreement with the reported reference method (28) was also tested. The results of the developed triplex qPCR showed more than 99.94% agreement for both positive and negative samples with those of the reference method (Table 7), and there was no significant difference between the results of these two methods using Chi-square-test analysis (*p* > 0.05).

**Table 7:** The agreement of the developed triplex qPCR with the reported reference method.

Detection method	Positive sample				Negative sample	Total
	ASFV (%)	Genotype I (%)	Genotype II (%)	Genotype I + II (%)		
The triplex qPCR	287/3,519 (8.16%)	6/3,519 (0.17%)	253/3,519 (7.19%)	28/3,519 (0.80%)	3232/3519 (91.84%)	3,519
The reported reference method (28)	286/3,519 (8.13%)	7/3,519 (0.20%)	251/3,519 (7.13%)	28/3,519 (0.80%)	3233/3519 (91.87%)	3,519
Agreement	99.97%	99.97%	99.94%	100%	99.97%	

Positive Percent Agreement (PPA): 100% (99.34–100% at 95% confidence interval). Negative Percent Agreement (NPA): 99.97% (99.86–100% at 95% confidence interval). Overall Percent Agreement (OPA): 99.97% (99.87–100% at 95% confidence interval).

The 3,519 clinical samples were also tested by the qPCR described in the Chinese national standard of *Diagnostic techniques for African swine fever* (GB/T 18648-2020). The positivity rate of this method for ASFV was 8.16% (287/3,519), which was consistent with the results of the developed triplex qPCR in this study.

## 4. Discussion

ASF is a highly contagious disease of domestic pigs and wild boars, and causes high morbidity rate up to 100% (3). Nowadays, ASF is still circulating in many countries and seriously impacts the swine industry around the world (8–10). Since 2018, ASF has been reported in many countries in Asia, and has caused serious economic losses to the pig industry in these countries (7, 17, 18). Genotypes I and II ASFV have been discovered in pig farms in China, and they caused different damages and manifestations to the infected pig herds (15, 30, 31). China is the largest pig breeding and pork production country in the world, and the outbreak of ASF in 2018 caused serious losses to Chinese pig industry (17, 18). At present, ASFV has become a common virus in Chinese pig herds, and the effective prevention and control of ASF is extremely important. Early detection and diagnosis, eradication of pathogenic positive pigs, and strict biosafety are important and effective measures to prevent and control ASF (32, 33). Development of methods for fast and accurate detection of genotypes I and II ASFV is very important to find the infected pigs in the early stage of infection. Therefore, a triplex qPCR was developed for specific, sensitive, and accurate detection of genotypes I and II ASFV in this study. The assay used the primers and probes based on the B646L, F1055L, and E183L genes for differential detection of genotype I, and genotype II ASFV, respectively. The assay showed high specificity, good sensitivity, and excellent repeatability. Then, a total of 3,519 clinical samples were used to evaluate the application of the developed method, and were validated by the duplex qPCR established by Cao et al. (28). The developed triplex qPCR showed a clinical sensitivity of 100% and a clinical specificity of 99.97%, and had more than 99.94% agreement with the reference method. In addition, these samples were also tested by the singleplex qPCR described in the Chinese national standard for detection of ASFV (GB/T 19648-2020), which was further used to validate the application of the developed assay, and the detection results of these two methods were completely consistent. These results indicated that the developed method has good application in the field.

Since ASFV was first discovered in Kenya in 1921, genotypes I and II ASFV have been reported in many countries around the world, with genotype II being responsible for the ongoing pig pandemic (7, 11, 12, 33–35). In this study, a total of 3,519 clinical samples were collected from Guangxi province in China during 2019 to 2023, and were tested by the developed triplex qPCR. As a result, the positivity rates of genotype I, genotype II, and genotypes I + II were 0.17, 7.19, and 0.80%, respectively, showing that genotype II strain was the predominant genotype in Guangxi province, but the genotype I strain was also circulating in pig herds. It is noteworthy that co-infection with genotypes I and II was found in 28 clinical samples, and this is an important factor that need to be considered when implementing the prevention and control measures for ASF. In addition, the positivity rates of harmless disposal sites, farmers markets, slaughterhouses, and pig farms were 25.72, 16.67, 1.50, and 1.02%, respectively (Table 5), indicating the high ASFV positivity rates of the samples from the harmless disposal sites, and farmers markets. This situation suggests that thorough cleaning, disinfection, and strict biosafety measures must be taken to prevent the spread of ASFV from these places.

Several assays have been reported to establish multiplex qPCR for differential detection of genotypes I and II ASFV (27–29). Li et al. used two pairs of primers and two probes targeting the E296R gene for differentiation of genotypes I and II ASFV, with the sensitivity of 10 copies/μL for both genotypes (27). Cao et al. used a pair of primers and two probes targeting the B646L gene for differentiation of two genotypes of ASFV, with the sensitivity of 10 copies/reaction for genotype I and 100 copies/reaction for genotype II, respectively (28). Gao et al. used two pairs of primers and two probes targeting the B646L gene of genotypes I and II, or the E183L gene of genotype I for differentiation of genotypes I and II ASFV, with the sensitivity of 1.07 × 10^2^ copies/μL for genotype I and 3.13 × 10^4^ copies/μL for genotype II (29). In our study, the specific primers and probes targeting the B646L gene for different genotypes, the F1055L gene for genotype I, and the F183L gene for genotype II were designed for detection and differentiation of genotypes I and II ASFV, with the sensitivity of 399.647 copies/reaction for B646L, 374.409 copies/reaction for F1055L, and 355.083 copies/reaction for E183L. All these assays were highly specific, sensitive, reproducible. Our assay resulted in similar sensitivity to the assay established by Li et al. (27), less sensitivity than the assay established by Cao et al. (28), and higher sensitivity than the assay established by Gao et al. (29). Therefore, the duplex qPCR established by Cao et al. (28) was used as a reference method to validate the developed triplex qPCR in this study. The results showed that the former method had similar positivity rate of ASFV (8.13%, 286/3,519) to the later method (8.16%, 287/3,519), and there no significant difference between them.

To date, only genotypes I and II ASFV have been reported outside Africa (12, 13). However, with the increase of travelers and trade in animals and animal products, it cannot be ruled out that other genotypes of ASFV might spread outside Africa to other countries and regions in the future. Therefore, three pairs of specific primers and three corresponding probes were designed in this study. The universal primers and probe targeting the B646L gene was used to detect all genotypes of ASFV, and the primers and probes targeting the F1055L gene or the E183L gene were used to detect and differentiate genotype I or genotype II. So, the developed assay could detect other genotypes besides genotype I and genotype II ASFV. On the contrary, the assays established by Li et al. (27) and Cao et al. (28) could only detect genotype I and genotype II, but not other genotypes; the assay established by Gao et al. (29) could detect genotype I and genotype II, but could not differentiate genotype I from genotype II in case of co-infection of both genotypes. The developed triplex qPCR in this study can simultaneously detect and distinguish genotype I and genotype II, without missing out other potential genotypes, which is the advantage of this method.

## 5. Conclusion

A triplex qPCR for detection and differentiation of genotypes I and II ASFV was developed in this study. The assay showed high sensitivity, excellent specificity and good repeatability. This assay provides an effective technical method for differential detection and epidemiological investigation of ASFV in the field. In addition, genotypes I and II ASFV were co-circulating in Guangxi province, southern China at present.

## Data availability statement

The raw data supporting the conclusions of this article will be made available by the authors, without undue reservation.

## Ethics statement

The animal studies were approved by Guangxi Center for Animal Disease Control and Prevention, China. The studies were conducted in accordance with the local legislation and institutional requirements. Written informed consent was obtained from the owners for the participation of their animals in this study.

## Author contributions

XQ: Investigation, Methodology, Writing – original draft. LH: Investigation, Methodology, Writing – original draft. KS: Funding acquisition, Writing – review & editing. HW: Investigation, Software, Validation, Writing – original draft. YS: Methodology, Software, Validation, Writing – original draft. XH: Investigation, Software, Validation, Writing – original draft. QZ: Investigation, Methodology, Writing – original draft. SF: Data curation, Supervision, Writing – review & editing. FL: Data curation, Methodology, Supervision, Writing – original draft. SM: Data curation, Methodology, Supervision, Writing – review & editing. ZL: Writing – review & editing.

## Footnotes

^1^ https://blast.ncbi.nlm.nih.gov/Blast.cgi

^2^ http://www.spc.net.cn
