# Supplementary material for: Development of a triplex real-time quantitative PCR for detection and differentiation of genotypes I and II African swine fever virus
Source: Front Vet Sci. 2023 Oct 19;10:1278714. doi: 10.3389/fvets.2023.1278714 (PMC10620837; doi:10.3389/fvets.2023.1278714)
Supplement: Supplementary file 1 [file Table_1.docx]

**Supplementary**

**TABLE S1 The sequences of the targeting fragments of 24 genotypes of ASFV**

| **Genotype** | **Reference Strain** | **Sequence** | **length/bp** |
| --- | --- | --- | --- |
| I(B646L) | Ang72  (FJ174378) | CAAAGTTCTGCAGCTCTTACATACCCTTCCACTACGGAGGCAATGCAATTAAAACCCCCGATGATCCGGGTGCGATGATGATTACCTTTGCTTTGAAGCCACGGGAGGAATACCAACCCA | 120 |
| II(B646L) | MOZ-60/98  (AY274455) | CAAAGTTCTGCAGCTCTTACATACCCTTCCACTACGGAGGCAATGCGATTAAAACCCCCGATGATCCGGGTGCGATGATGATTACCTTTGCTTTGAAGCCACGGGAGGAATACCAACCCA | 120 |
| III(B646L) | BOT/1/99  (AF504886) | CAAAGTTCTGCAGCTCTTACATACCCTTCCACTACGGAGGCAATGCGATTAAAACCCCCGACGATCCGGGTGCGATGATGATTACCTTTGCTTTGAAGCCACGGGAGGAATACCAACCCA | 120 |
| IV(B646L) | RSA/1/99/W (AF449477) | CAAAGTTCTGCAGCTCTTACATACCCTTCCACTACGGAGGCAATGCGATTAAAACCCCCGACGATCCGGGTGCGATGATGATTACCTTTGCTTTGAAGCCACGGGAGGAATACCAACCCA | 120 |
| V(B646L) | Teng ani (AF301541) | CAAAGTTCTGCAGCTCTTACATACCTTTCCACTACGGAGGCAATGCGATTAAAACCCCCGATGATCCGGGTGCGATGATGATTACCTTTGCCTTGAAGCCACGGGAGGAATACCAACCCA | 120 |
| VI(B646L) | SPEC265 (AF270710) | CAAAGTTCTGCAGCTCTTACATACCTTTCCACTACGGAGGCAATGCGATTAAAACCCCCGACGATCCGGGTGCGATGATGATTACCTTTGCCTTGAAGCCACGGGAGGAATACCAACCCA | 120 |
| VII(B646L) | SPEC/260 DQ250121 | CAAAGTTCTGCAGCTCTTACATACCCTTCCACTACGGAGGCAGTGCGATTAAAACCCCCGACGATCCGGGTGCGATGATGATTACCTTTGCTTTGAAGCCACGGGAGGAATACCAACCCA | 120 |
| VIII(B646L) | THY/901 AY351545 | CAAAGTTCTGCAGCTCTTACATACCCTTCCACTATGGAGGCAATTCGATTAAAACCCCCGATGATCCGGGCGCGATGATGATTACCTTTGCTTTGAAGCCACGGGAGGAATACCAACCCA | 120 |
| IX(B646L) | Ug03H.1 (FJ154428) | CAAAGTTCTGCAGCTCTTACATACCCTTCCACTACGGAGGCAATTCGATTAAAACCCCCGACGATCCGGGCGCGATGATGATTACCTTTGCTTTGAAACCACGGGAGGAATACCAACCCA | 120 |
| X(B646L) | UGA/3/95 (AF449476) | CAAAGTTCTGCAGCTCTTACATACCCTTTCACTACGGAGGCAATTCGATTAAAACCCCCGACGATCCGGGCGCGATGATGATTACCTTTGCTTTGAAACCACGGGAGGAATACCAACCCA | 1201 |
| XI(B646L) | KAB/62 (AY351522) | CAAAGTTCTGCAGCTCTTACATACCCTTCCACTACGGAGGCAATTCGATTAAAACCCCCGACGATCCGGGCGCGATGATGATTACCTTTGCTTTGAAGCCACGGGAGGAATACCAACCCA | 120 |
| XII(B646L) | MZI/921 （AY351543） | CAAAGTTCTGCAGCTCTTACATACCCTTCCACTACGGAGGCAATTCGATTAAAACCCCCGACGATCCGGGCGCGATGATGATTACCTTTGCTTTGAAGCCACGGGAGGAATACCAACCCA | 120 |
| XIII(B646L) | SUM/1411 （AY351542） | CAAAGTTCTGCAGCTCTTACATACCCTTCCACTACGGAGGCAATTCGATTAAAACCCCCGACGATCCGGGCGCGATGATGATTACCTTTGCTTTGAAGCCACGGGAGGAATACCAACCCA | 120 |
| XIV(B646L) | NYA/12 （AY351555） | CAAAGTTCTGCAGCTCTTACATACCCTTCCACTACGGAGGTAATTCGATTAAAACTCCCGACGATCCGGGCGCGATGATGATTACCTTT  GCTTTGAAGCCACGGGAGGAATACCAACCCA | 120 |
| XV(B646L) | TAN/1/01 （AY494552 ） | CAAAGTTCTGCAGCTCTTACATACCCTTCCACTACGGAGGCAATTCGATTAAAACTCCTGACGATCCGGGCGCGATGATGATTACCTTT  GCTTTGAAGCCACGGGAGGAATACCAACCCA | 120 |
| XVI(B646L) | TAN/2003/1 （AY494550） | CAAAGTTCTGCAGCTCTTACATACCCTTCCACTACGGAGGCAGTTCGATTAAAACTCCTGACGATCCGGGTGCGATGATGATTACCTTT  GCTTTGAAGCCACGGGAGGAATACCAACCCA | 120 |
| XVII(B646L) | ZIM/92/1 (DQ250119) | CAAAGTTCTGCAGCTCTTACATACCCTTCCACTACGGAGGCAATGCGATTAAAACCCCCGATGATCCGGGTGCGATGATGATTACCTTT  GCTTTGAAGCCACGGGAGGAATACCAACCCA | 120 |
| XVIII(B646L) | NAM/1/95 (DQ250122) | CAAAGTTCTGCAGCTCTTACATACCCTTCCACTACGGAGGCAATGCGATTAAAACCCCCGATGATCCGGGTGCGATGATGATTACCTTTGCTTTGAAGCCACGGGAGGAATACCAACCCA | 120 |
| XIX(B646L) | RSA/2/96 (DQ250126) | CAAAGTTCTGCAGCTCTTACATACCCTTCCACTACGGAGGCAATGCGATTAAAACCCCCGACGATCCGGGCGCGATGATGATTACCTTTGCTTTGAAGCCACGGGAGGAATACCAACCCA | 120 |
| XX(B646L) | RSA/1/95 (DQ250123) | CAAAGTTCTGCAGCTCTTACATACCCTTCCACTACGGAGGCAATGCGATTAAAACCCCCGACGATCCGGGTGCGATGATGATTACCTTT  GCTTTGAAGCCACGGGAGGAATACCAACCCA | 120 |
| XXI(B646L) | RSA/1/96 (DQ250125) | CAAAGTTCTGCAGCTCTTACATACCCTTCCACTACGGAGGCAATGCGATTAAAACCCCCGACGATCCGGGTGCGATGATGATTACCTTT  GCTTTGAAGCCACGGGAGGAATACCAACCCA | 120 |
| XXII(B646L) | SPEC/245 (DQ250117) | CAAAGTTCTGCAGCTCTTACATACCCTTCCACTACGGAGGCAATGCGATTAAAACCCCTGACGATCCGGGTGCGATGATGATTACCTTT  GCTTTGAAGCCACGGGAGGAATACCAACCCA | 120 |
| XXIII(B646L) | ETH/3 (KT795360) | CAAAGTTCTGCAGCTCTTACATACCCTTCCACTACGGAGGCAATTCGATTAAAACCCCCGACGATCCGGGTGCGATGATGATTACCTTT  GCTTTGAAGCCACGGGAGGAATACCAACCCA | 120 |
| XXIV(B646L) | MOZ_16/2006 (KY353995) | CAAAGTTCTGCAGCTCTTACATACCCTTCCACTACGGAGGCAGTGCGATTAAAACCCCCGACGATCCGGGTGCGATGATGATTACCTTT  GCTTTGAAGCCACGGGAGGAATACCAACCCA | 120 |
| I (F1055L) | BA71V  (KP055815) | GCAGGTAGTTTGATTCCCTTTGTGAGACAGCAGATTAAGCAGAGCCCCTGCGTTGTTTCAAACCTCTTGCCCATAACAGAGACTTCCTCAGTAAAGGAGGAGACACTTACAGAGACATCGCCC | 122 |
| II (E183L） | Wuhan 2019-2  (MN393477) | CGCGAGTGCTCCTGCTCATCCGGCTGAGCCTTACACGACAGTCACTACTCAGAACACTGCTTCACAAACAATGTCGGCTATTGAAAATTTACGACAAAGAAACACCTATACGCATAAAGACCTAGAAAACTCC | 133 |
